# Using Machine Learning to Identify the Relationships between Demographic, Biochemical, and Lifestyle Parameters and Plasma Vitamin D Concentration in Healthy Premenopausal Chinese Women

**DOI:** 10.3390/life13122257

**Published:** 2023-11-27

**Authors:** Chun-Kai Wang, Ching-Yao Chang, Ta-Wei Chu, Yao-Jen Liang

**Affiliations:** 1 Department of Obstetrics and Gynecology, Zuoying Branch of Kaohsiung Armed Forces General Hospital, Kaohsiung 813, Taiwan; a0978060003@mail.ngh.com.tw; 2 Graduate Institute of Applied Science and Engineering, Fu Jen Catholic University, New Taipei City 242, Taiwan; 411068065@m365.fju.edu.tw; 3 Department of Obstetrics and Gynecology, Tri-Service General Hospital, National Defense Medical Center, Chief Executive Officer’s Office, MJ Health Research Foundation, Taipei 114, Taiwan; david_chu@mjlife.com

**Keywords:** machine learning, premenopausal women, vitamin D

## Abstract

Introduction: Vitamin D plays a vital role in maintaining homeostasis and enhancing the absorption of calcium, an essential component for strengthening bones and preventing osteoporosis. There are many factors known to relate to plasma vitamin D concentration (PVDC). However, most of these studies were performed with traditional statistical methods. Nowadays, machine learning methods (Mach-L) have become new tools in medical research. In the present study, we used four Mach-L methods to explore the relationships between PVDC and demographic, biochemical, and lifestyle factors in a group of healthy premenopausal Chinese women. Our goals were as follows: (1) to evaluate and compare the predictive accuracy of Mach-L and MLR, and (2) to establish a hierarchy of the significance of the aforementioned factors related to PVDC. Methods: Five hundred ninety-three healthy Chinese women were enrolled. In total, there were 35 variables recorded, including demographic, biochemical, and lifestyle information. The dependent variable was 25-OH vitamin D (PVDC), and all other variables were the independent variables. Multiple linear regression (MLR) was regarded as the benchmark for comparison. Four Mach-L methods were applied (random forest (RF), stochastic gradient boosting (SGB), extreme gradient boosting (XGBoost), and elastic net). Each method would produce several estimation errors. The smaller these errors were, the better the model was. Results: Pearson’s correlation, age, glycated hemoglobin, HDL-cholesterol, LDL-cholesterol, and hemoglobin were positively correlated to PVDC, whereas eGFR was negatively correlated to PVDC. The Mach-L methods yielded smaller estimation errors for all five parameters, which indicated that they were better methods than the MLR model. After averaging the importance percentage from the four Mach-L methods, a rank of importance could be obtained. Age was the most important factor, followed by plasma insulin level, TSH, spouse status, LDH, and ALP. Conclusions: In a healthy Chinese premenopausal cohort using four different Mach-L methods, age was found to be the most important factor related to PVDC, followed by plasma insulin level, TSH, spouse status, LDH, and ALP.

## 1. Introduction

Vitamin D plays a crucial role in maintaining homeostasis and promoting the absorption of calcium, an essential component for strengthening bones and preventing osteoporosis [1,2]. It is a pro-hormone that, in the classical pathway, is activated by sequential hydroxylation at C25 and C1 to produce 1,25(OH)2D3, which is biologically active and acts predominantly on the vitamin D receptors in the classical pathway [3]. In addition, new pathways of vitamin D activation by CYP11A1 were established, describing the production of several biologically active hydroxyderivatives [4,5,6,7,8], acting on different nuclear receptors in addition to the vitamin D receptors [9]. While severe vitamin D deficiency is rare, it can lead to rickets in children and osteomalacia in adults [1]. On the other hand, a widespread subclinical deficiency of this vitamin is linked to osteoporosis, increasing the risk of falls and fractures [2]. Apart from its primary function in calcium metabolism, vitamin D receptors are present in various organs and tissues, suggesting its potential impact on multiple biological processes. Research indicates that vitamin D deficiency may contribute to the progression of conditions such as tuberculosis [10], respiratory tract infections [11], asthma [12], and atopic dermatitis [13], as it influences both innate and adaptive immunity. Furthermore, studies have documented increased risks of hypertension, cardiovascular diseases, cancer, musculoskeletal pain, and migraines associated with vitamin D insufficiency [14,15,16,17].

Vitamin D can be obtained efficiently through various means, including dietary consumption, exposure to sunlight, or supplementation. Specific guidelines for vitamin D supplementation may vary, taking into account factors such as age, health conditions, and individual considerations. Research has shown that vitamin D deficiency is prevalent in the general population. According to the 2013 National Health and Nutrition Examination Survey, approximately 70% of women are reported to experience this deficiency [18]. Similarly, the National Nutrition Survey, conducted from 2006 to 2008 and involving 2,596 participants aged 19 and above, revealed that 66.2% of individuals had inadequate vitamin D levels [19]. Surprisingly, even regions known for high sunlight exposure, like the southern United States, show a significant incidence of vitamin D deficiency [20]. Adequate supplementation of vitamin D is essential to prevent various diseases, improve prognosis, and maintain proper cellular functioning in organs. Therefore, understanding the factors that influence the concentration of vitamin D in the bloodstream of the general population is of significant interest.

Before the era of artificial intelligence developed, most of the studies used multiple linear regression (MLR) to evaluate the relationships between independent variables and dependent variables. It should be noted that both variables should be continuous. MLR could adjust the confounding effects between variables. However, with the recent emergence of artificial intelligence, machine learning (Mach-L) has become a powerful alternative. Unlike MLR, Mach-L enables machines to learn from past data or experiences without explicit programming. Moreover, it could capture non-linear interactions between complicated variables, including continuous, ordinal, and categorical variables, which makes it a strong competitor in the medical research field [21,22,23].

One of the key advantages of Mach-L is its ability to understand non-linear relationships and complex interactions among multiple predictors. This capability positions it to excel over traditional MLR in disease prediction [24]. As a result, Mach-L offers promising potential for advancing research in understanding and predicting conditions, like vitamin D deficiency, and their associated risk factors.

To the best of our knowledge, no previous study has explicitly examined the associations between plasma 25-OH vitamin D concentration (PVDC) and demographic, biochemical, and lifestyle factors using machine learning (Mach-L) techniques. In this research, we recruited participants from a health-checkup chain clinic with two main objectives: (1) to assess and compare the predictive accuracy of Mach-L and MLR, and (2) to establish a hierarchy of the significance of factors—including demographic, biochemical, and lifestyle aspects—in relation to the plasma concentrations of PVDC.

## 2. Methods

### 2.1. Participants and Study Design

**Figure 1:**
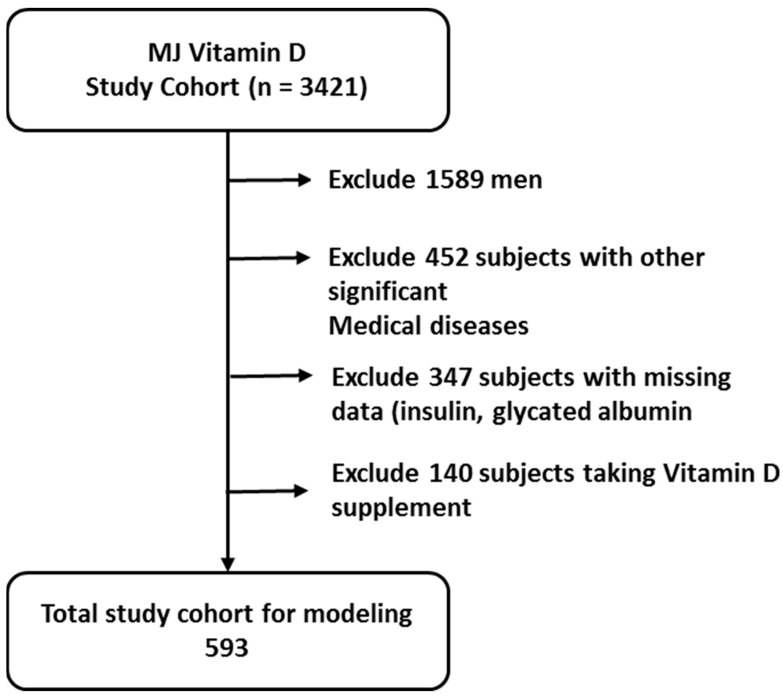
Participant selection scheme.

The data for this study were obtained from the MJ Health Screening Center, a private clinical chain with three centers located in northern, central, and southern Taiwan. The MJ Health Database only comprises individuals who have given informed consent. All or part of the data used in this research were authorized by and received from the MJ Health Research Foundation (authorization code: MJHRF2022011A). Any interpretations or conclusions described in this paper are those of the authors alone and do not represent the views of the MJ Health Research Foundation. Initially, a total of 1532 healthy women were included. After excluding subjects with missing data, those taking vitamin D supplements at the time of the study, and individuals with significant medical diseases, the final analysis comprised 593 women between 20 and 50 years old (Figure 1). The main reason to select this age range was to exclude participants with menopause. The criteria for participant inclusion can be found in Table 1. Prior to their routine health examination, all participants provided informed consent, and the collected data were anonymized. The study protocol was approved by the institutional review board of the Zuoying Branch of Kaohsiung Armed Forces General Hospital (IRB No. KAFGHIRB 110-23). Participants with serious health conditions, such as cancer, were not included in the study.

Throughout the study, a senior nursing staff member took a comprehensive record of the participants’ medical history, including information about their current medications. The status of education level, family income, having a spouse, drinking alcohol, having betel nuts, smoking, daily sport hours, and sleep hours were also recorded.

### 2.2. Proposed Mach-L Scheme

The following description of the methods related to Mach-L was published in our previous work [25]. This research proposes a scheme based on four machine learning (Mach-L) methods: random forest (RF), stochastic gradient boosting (SGB), extreme gradient boosting (XGBoost), and elastic net. The primary objective is to construct predictive models for forecasting plasma vitamin D levels and identify the significance of related risk factors. These Mach-L methods, widely used in various healthcare applications, are advantageous as they do not make prior assumptions about data distribution [26,27,28,29,30,31,32,33,34,35]. For comparison, multiple linear regression (MLR) was used as the reference.

The first method, random forest (RF), is an ensemble learning decision tree algorithm that combines bootstrap resampling and bagging [36]. RF generates multiple random and unpruned CART decision trees using the decrease in Gini impurity as the splitting criterion. These trees are then assembled into a forest, and their predictions are averaged or voted upon to generate output probabilities and a final model, providing robust predictions [37].

The second method, stochastic gradient boosting (SGB), is a tree-based gradient boosting learning algorithm that combines bagging and boosting techniques to minimize the loss function and mitigate overfitting issues encountered in traditional decision trees [38,39]. Through multiple iterations, SGB generates a series of stochastic weak learners in the form of trees. Each tree aims to correct or explain the errors made by the preceding tree. The residual of the previous tree serves as input for the newly generated tree. This iterative process continues until a convergence condition or stopping criterion is met. The final robust model is determined by the cumulative results of these trees.

Thirdly, extreme gradient boosting (XGBoost) is an optimized extension of SGB based on gradient boosting technology [40]. It trains numerous weak models sequentially and combines them using the gradient boosting method, resulting in improved prediction performance. XGBoost employs Taylor binomial expansion to approximate the objective function and differentiable loss functions to expedite the model’s construction convergence process [41]. Additionally, XGBoost applies regularized boosting techniques to penalize model complexity and address overfitting, thereby enhancing the overall model accuracy [40].

The final method is elastic net regression, which is a linear regression technique that incorporates a penalty term to shrink the coefficients of the predictors. This penalty term is a combination of the l1-norm (absolute value) and the l2-norm (square) of the coefficients, weighted by a parameter called alpha. The l1-norm penalty, similar to lasso regression, tends to produce sparse solutions by setting some coefficients to zero. On the other hand, the l2-norm penalty, similar to ridge regression, aims to reduce the variance of the coefficients by shrinking them toward zero. By combining the strengths of both lasso and ridge, elastic net regression can handle situations where there are correlated predictors and potentially improve the model’s predictive performance [42].

**Figure 2:**
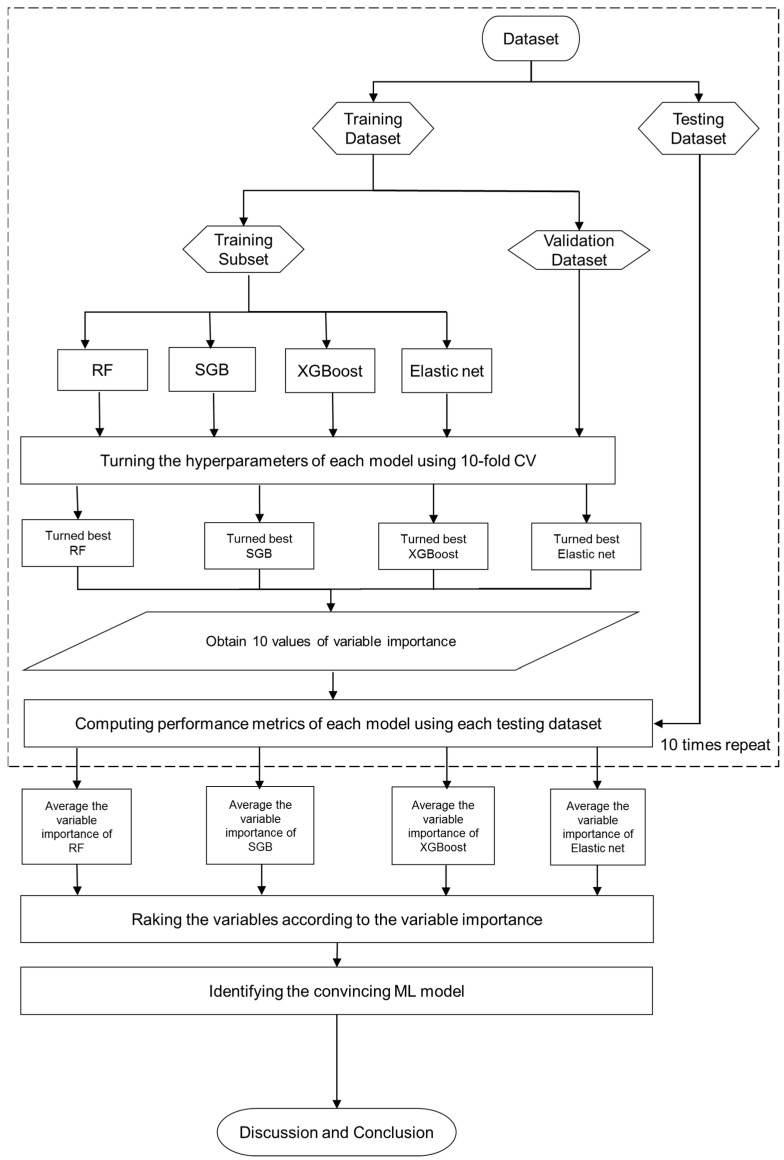
Proposed machine learning prediction scheme.

Figure 2 depicts the flowchart of the proposed prediction and significant variable identification scheme that integrates the four Mach-L methods. The process begins with the collection of patient data and the preparation of the dataset using the proposed method. Subsequently, the dataset is randomly split into an 80% training dataset for model building and a 20% testing dataset for model evaluation. During the training process, each Mach-L method involves specific hyperparameters that necessitate tuning to construct high-performing models. For this tuning, a 10-fold cross-validation technique is employed. The training dataset is further divided into a training set, where various sets of hyperparameters are used for model construction, and a validation set for model validation. A grid search explores all possible combinations of hyperparameters, and the model exhibiting the lowest root mean square error for the validation dataset is selected as the best model for each Mach-L method. Consequently, the best-tuned models for RF, SGB, XGBoost, and elastic net are generated, along with the variable importance ranking information. To determine the significance of variables, the importance ranks from these four methods are averaged, yielding the results of their importance.

During the testing process, the testing dataset is employed to evaluate the predictive performance of the best RF, SGB, and elastic net models. As the target variable in this study is numerical, several metrics are used for model performance comparison, including mean absolute percentage error (MAPE), symmetric MAPE (SMAPE), relative absolute error (RAE), root relative squared error (RRSE), and root mean square error (RMSE). The equations for these performance metrics are provided in Table 2.

### 2.3. Statistical Methods

The Kolmogorov–Smirnov test was employed to assess the normal distribution of the data, while Levene’s test was utilized to check the homogeneity of the variances. Continuous variables were represented as the mean plus or minus the standard deviation. An independent t-test was used to analyze PVDC in subjects with or without a spouse. For other ordinal variables, such as sleep hours, education level, income, and smoking, a one-way analysis of variance was applied. For evaluating the relationships between PVDC and other continuous variables, Pearson’s correlation was utilized. All statistical analyses were conducted using version 13.0 of the SPSS software system (SPSS Inc., Chicago, IL, USA). All p-values less than 0.05 were deemed statistically significant.

## 3. Results

In total, there were 593 participants enrolled in this study. The mean age was 37.98 ± 7.58 years old, and the body fat percentage was 29.65 ± 7.42%. The mean and standard deviation of 35 variables and their corresponding units are shown in Table 1. It should be noted that PVDC was significantly higher in subjects with spouses compared to their counterparts. At the same time, there was no significant difference in PVDC between subjects with different sleep hours, education levels, and family incomes.

Table 3 shows the result of Pearson’s correlation between risk factors and PVDC. It could be noted that age, HDL-cholesterol, LDL-cholesterol, and hemoglobin were positively correlated with PVDC. At the same time, all other factors were not significantly correlated with PVDC.

Table 4 shows the model performance of the MLR, RF, SGB, XGBoost, and elastic net. The MAPE, SMAPE, RAE, RRSE, and RMSE values of RF, SGB, XGBoost, and elastic net were all smaller than those of the MLR. This indicates that these Mach-L methods were more accurate compared to MLR. 

**Figure 3:**
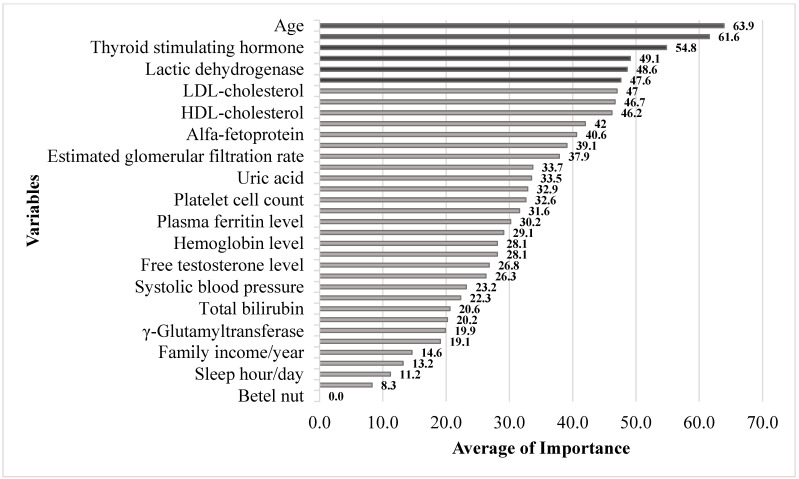
The means of importance of the risk factors derived from four different machine learning methods.

In Table 5, the variables of importance, their means, and the mean rank of importance are displayed. From this table, age was the most important factor, followed by plasma insulin level, TSH, spouse status, LDH, and ALP. The graphic illustration of these variables and their importance is shown in Figure 3.

## 4. Discussion

In the present study, we employed four different Mach-L methods to identify six parameters that are significantly related to plasma vitamin D levels (PVDL) in healthy Chinese women aged 20–53 years old. As mentioned in the introduction, Mach-L techniques are capable of capturing non-linear relationships, making them valuable tools for medical research across various domains. While some previous studies have used Mach-L to explore factors influencing vitamin D, most of them have focused on diagnosing vitamin D deficiency, and as a result, statistical and Mach-L methods dealing with binary variables, such as MLR, were predominantly used. For example, Sancar et al. performed a study on 481 subjects [43]. Four different Mach-L methods, namely, ordinal logistic regression (OLR), elastic-net ordinal regression (ENOR), support vector machine (SVM), and random forest (RF), were compared. They concluded that the accuracy of SVM was significantly and negatively influenced when the method was examined. At the same time, RF was the most robust among these four methods when the size of the training set varied. The accuracy, sensitivity, precision, F1-score, and Cohen’s kappa were further provided, and they were all higher than 0.9. From their findings, they suggested that RF was a potential better tool to detect vitamin D levels and could be used in routine clinical settings. It is interesting to note that the discussion of this article mainly focused on the details of Mach-L methods such as parameter tuning, sensitivities to decreasing sample sizes, and classification performance. Little was emphasized about which one of the variables (demographic, biochemical, and lifestyle details) used was more clinically relevant to vitamin D concentrations. Thus, even though the authors had shown that Mach-L methods were accurate enough to be used, it would not be possible for medical providers to use these methods practically. In fact, many of these studies were more closely related to the engineering and mathematics fields. In contrast, our study focuses on predicting vitamin D levels using Mach-L and identifying significant factors in a healthy population of Chinese women within a specific age range [44,45]. Our study is the first to use Mach-L methods to set PVDC as a continuous variable. Moreover, the present study included demographic, biochemical, and lifestyle information, whose importance, to the best of our knowledge, has never been previously reported at the same time.

The most crucial factor identified by Mach-L in the present study was age, which showed a positive correlation with PVDC. This finding differs from most other studies that have shown a decrease in PVDC with increasing age. However, two major underlying pathophysiological mechanisms may explain this relationship. First, an age-related decline in renal function leads to a 50% reduction in the production of 1,25(OH)2D. Second, a decrease in calcium absorption in aged individuals occurs before the decline in 1,25(OH)2D by approximately 10 to 15 years. These factors may contribute to the observed positive correlation between age and PVDC in our study [46,47,48]. However, in contrast to our findings, other studies have reported opposite results. The conflicting results in the literature are not entirely surprising and can be attributed to two main reasons. Firstly, the concentration of PVDC may vary significantly among different ethnic groups. Secondly, most other studies did not separate genders and included all age groups, which might introduce confounding factors. Therefore, further studies with more sophisticated classifications and larger sample sizes are needed to better understand the relationships between age and PVDC in different populations.

The second factor identified by Mach-L in the present study was plasma insulin level. However, it is essential to note that there is limited research on the direct relationship between PVDC and plasma insulin levels. Most previous studies have primarily focused on the potential improvement of insulin resistance after vitamin D supplementation. This improvement is believed to occur through the effects of vitamin D on muscle cell receptors. Vitamin D can increase insulin receptor expression or enhance the sensitivity of insulin receptors to insulin. It is well established that individuals with insulin resistance tend to have higher insulin levels. Therefore, vitamin D’s impact on insulin receptors and related pathways may contribute to the observed association between plasma insulin level and PVDC in our study [49]. The decrease in insulin resistance typically leads to a reduction in plasma insulin and glucose levels following vitamin D supplementation, as observed in previous studies. Conversely, there is evidence to suggest that vitamin D can stimulate insulin secretion directly through its receptors or indirectly by regulating intracellular calcium levels to facilitate insulin secretion [50,51]. These findings align with our present study results, indicating that vitamin D may have a positive impact on insulin levels. Moreover, indirect evidence also supports the increase in insulin levels seen in our study. For individuals with vitamin D deficiency, supplementation with vitamin D may reduce the incidence of type 2 diabetes, further supporting the potential beneficial effect of vitamin D on insulin levels.

In the past, researchers were interested in the complex molecular interactions between vitamin D and thyroid function. It has been noted that patients with hypothyroidism have a higher chance of having low levels of vitamin D. The underlying cause for this phenomenon was postulated due to the strong similarity between the two receptors of vitamin D3 and thyroid hormone since they evolved from a single primordial gene [52,53]. The synthesis of 1,25 dihydroxy-vitamin D or calcitriol, the active vitamin D metabolites, all depends on the enzyme 1-alpha hydroxylase, which is mainly expressed in the kidney [54]. These pieces of evidence support the result of the present study, where a negative correlation was found between PVDC and TSH levels.

The next factor found in the present study was whether the participant had a spouse, and, in this cohort, participants with a spouse were defined as ‘not living alone’. It is intriguing to note that there was a significant difference in PVDC between those with a spouse and those without (22.0 ± 7.6 versus 19.1 ± 7.36, respectively). This relationship has rarely been reported in the literature, and to our knowledge, only one other study has reached a similar conclusion. Khalfa et al. demonstrated that vitamin D3 levels were significantly lower in single women compared to their counterparts. While this finding could be partially explained by sexual activity, these studies only provided indirect evidence. In support of our present study’s results, a study by Canguven and colleagues [55] showed that vitamin D treatment improved the sexual activity of men. This is closely related to what we observed in our study, where single females were more likely to have deficient vitamin D3 levels due to a potential lack of sexual activity and the interplay of hormones. Furthermore, another study by Kidir [56] showed that sexual dysfunction in dialysis patients improved after vitamin D treatment, providing additional support for the potential role of vitamin D in sexual function. Although these findings offer valuable insights, further studies with more detailed designs are needed to explore the precise role of marital status and its association with PVDC.

The fifth factor chosen by Mach-L was plasma LDH concentration. LDH is generally considered a marker for inflammation [57]. At the same time, vitamin D has been proven to have anti-inflammatory effects [58]. These pieces of evidence contradict the results of our present study. Our data showed a non-significant but positive correlation between PVDC and LDH levels in a Pearson’s correlation analysis. At present, it is challenging to explain this discrepancy. Several factors might contribute to this disparity. Firstly, the fact that only healthy young women were enrolled in our study could have influenced the relationship between PVDC and LDH levels. It is possible that this specific population’s unique characteristics may have affected the correlation between the two variables. Secondly, differences in ethnic groups could also play a role in the observed discrepancy. Genetic and physiological variations among ethnic populations may impact the associations between PVDC and LDH levels.

Kover et al. were pioneers in establishing alkaline phosphatase (ALP) as a marker for vitamin D3 deficiency in premature infants [59]. Subsequently, numerous other studies have provided further support for the negative relationships between ALP levels and vitamin D3 levels [60,61,62]. It is important to note that only one study has been conducted in a diverse age group ranging from 10 to 80 years old. However, this study had a relatively small sample size, enrolling only 110 subjects, and did not find any significant correlation between ALP and PVDC [63]. Elevated serum levels of ALP are often indicative of increased bone turnover, and some researchers and clinicians consider it a bone formation marker [64]. This relationship is particularly strong in patients with osteomalacia [65], which could provide an explanation for the results of our present study. Our finding, which utilized Mach-L methods and included a larger cohort of 593 women, could further contribute to understanding this complex relationship.

It is interesting to note that either LDL-C or HDL-C were not selected by Mach-L in the present study. This is in contrast with others’ findings. For example, Li et al. reported that total cholesterol, LDL-C, and TG decreased if vitamin D concentration increased [66]. These relationships could be explained by the recently found roles of liver X receptors (LXRs). LXR is a nuclear receptor for oxysterol, which is an oxygenated derivative of cholesterol. At the same time, LXR is also a nuclear receptor for 1,25(OH)2D3 and 20,23(OH)2D3. Further studies are needed in the future to explore these relationships [67].

The present study still has limitations. First, this is a cross-sectional study, which is less solid than a longitudinal study. Secondly, this study was performed on only one ethnic group. Extrapolation to other ethnic groups should be exercised with caution. Further studies with a longitudinal design and a larger cohort are needed to elucidate the influencers of PVDC.

## 5. Conclusions

By using four different Mach-L methods, the six most important factors were selected. Age was the most important one, followed by plasma insulin level, TSH, having a spouse, LDH, and ALP in a group of healthy Chinese premenopausal women.

## Figures and Tables

**Table 1:** Demographic, biochemical, and lifestyle data of participants.

Variable		Values
n		593
Age (year)		37.98 ± 7.58
Body fat percentage (%)		29.65 ± 7.42
Systolic blood pressure (mmHg)		108.5 ± 13.87
Diastolic blood pressure (mmHg)		70.92 ± 9.86
Fasting plasma glucose (mg/dL)		95.14 ± 7.72
Glycated hemoglobin (%)		5.3 ± 0.37
Plasma insulin level (μU/mL)		6.67 ± 3.65
Triglyceride (mg/dL)		77.1 ± 41.28
HDL-cholesterol (mg/dL)		65.08 ± 14.15
LDL-cholesterol (mg/dL)		109.34 ± 30.4
Hemoglobin (g/dL)		13.06 ± 1.29
Platelet cell count (*10^3^/μL)		249.46 ± 58.51
White blood cell count (*10^3^/μL)		5.95 ± 1.56
Alkaline phosphatase (IU/L)		50.02 ± 15.78
Glutamic oxaloacetic transaminase (IU/L)		20.42 ± 7.29
Glutamic pyruvic transaminase (IU/L)		20.43 ± 16.48
Total bilirubin (mg/dL)		0.9 ± 0.31
γ-Glutamyltransferase (IU/L)		19.16 ± 13.24
Plasma calcium level (mg/dL)		9.59 ± 0.36
Plasma ferritin level (μg/dL)		82.1 ± 35.97
Plasma phosphate level (mg/dL)		3.88 ± 0.47
Uric acid (mg/dL)		4.71 ± 1.06
Alfa-fetoprotein (ng/mL)		3.15 ± 10.52
Carcinoembryonic antigen (ng/mL)		1.34 ± 0.74
Estimated glomerular filtration rate (mL/min/1.73m2)		86.54 ± 12.63
Lactic dehydrogenase (IU/L)		150.41 ± 23.64
High-sensitivity C-reactive protein (mg/L)		1.4 ± 2.28
Forced expiratory volume in one second (L)		93.52 ± 15.07
Thyroid-stimulating hormone (uIU/mL)		1.75 ± 1.03
Free-testosterone level (pg/mL)		3.53 ± 1.93
25-OH vitamin D (ng/mL)		20.86 ± 7.69
Exercise hour		7.58 ± 8.29
With or without spouse		
Single	211 (38.86)	19.1 ± 7.4 *
With spouse	332 (61.14)	22.0 ± 7.6
Sleep hours		
0–4 h/day	7(1.25)	21.6 ± 9.1
4–6 h/day	147 (26.25)	21.1 ±7.6
6–7 h/day	267 (47.68)	20.2 ± 7.5
7–8 h/day	113 (20.18)	18.9 ± 6.7
8–9 h/day	23 (4.11)	19.1 ±4.4
>9 h/day	3 (0.54)	21.0 ± 5.0
Education level		
Junior high school	9 (1.68)	21.4 ± 8.4
Senior high school	82 (15.27)	22.1 ± 7.2
College	97 (18.06)	21.5 ± 7.9
University	266 (49.53)	20.5 0 7.9±
Higher than master degree	83 (15.46)	20.4 ±6.8
Family Income (thousand USD/year)		
<6.1/year	87 (16.93)	26.6 ± 9.1
<6.1–12.1/year	128 (24.90)	21.1 ± 7.6
12.1–24.2/year	175 (34.05)	20.2 ± 2.5
24.2–36.2/year	79 (15.37)	21.4 ± 7.4
36.2–48.3/year	22 (4.28)	18.9 ± 6.6
48.3–60.4/year	12 (2.33)	19.2 ± 4.4
>60.4/year	11 (2.14)	210 ± 5.0

* *p* < 0.001.

**Table 2:** Equations of performance metrics.

Metrics	Description	Calculation
SMAPE	Symmetric Mean Absolute Percentage Error	$SMAPE=\frac{1}{n}\sum_{i=1}^{n}\frac{\left|y_{i}-{\hat{y}}_{i}\right|}{\left(\left|y_{i}\right|+\left|{\hat{y}}_{i}\right|\right)/2}\times 100$
RAE	Relative Absolute Error	$RAE=\sqrt{\frac{\sum_{i=1}^{n}{\left(y_{i}-{\hat{y}}_{i}\right)}^{2}}{\sum_{i=1}^{n}{\left(y_{i}\right)}^{2}}}$
RRSE	Root Relative Squared Error	$RRSE=\sqrt{\frac{\sum_{i=1}^{n}{\left(y_{i}-{\hat{y}}_{i}\right)}^{2}}{\sum_{i=1}^{n}{\left(y_{i}-{\hat{y}}_{i}\right)}^{2}}}$
RMSE	Root Mean Squared Error	$RMSE=\sqrt{\frac{1}{n}\sum_{i=1}^{n}{\left(y_{i}-{\hat{y}}_{i}\right)}^{2}}$

**Table 3:** The results of Pearson’s correlation between baseline demographic, biochemical, lifestyle, and δ-T score.

Age	Body Fat	SBP	DBP	HbA1c	PI	TG	HDL-C	LDL-C	Hb	Platelet	WBC	ALP	GOT	GPT	TB	GGT	Ca	P	Fe	UA	AFP	CEA	LDH	Hs-CRP	TSH	T
0.187 **	−0.032	0.045	0.052	0.113	−0.030	0.058	0.090 *	0.099 *	0.094 *	0.009	−0.016	−0.012	−0.026	−0.057	0.041	0.019	0.073	0.083	0.064	0.047	−0.017	0.073	0.074	−0.006	−0.075	−0.081

SBP: systolic blood pressure, DBP: diastolic blood pressure, PI: plasma insulin level, HDL-C: high-density lipoprotein cholesterol, LDL-C: low-density lipoprotein cholesterol, Hb: hemoglobin, WBC: white blood cell count, ALP: alkaline phosphatase, GOT: glutamic oxaloacetic transaminase, GPT: glutamic pyruvic transaminase, TB: total bilirubin, GGT: γ-glutamic transferase, Ca: plasma calcium level, P: plasma phosphate level, Fe: ferritin, UA: uric acid, AFP: a-fetoprotein, CEA: carcinoembryonic antigen, LDH: lactate dehydrogenase, Hs-CRP: high-sensitivity C-reactive protein, TSH: thyroid-stimulating hormone, T: plasma testosterone level. * *p* < 0.05; ** *p* < 0.001.

**Table 4:** The average performance of linear regression and four machine learning methods.

	MAPE	SMAPE	RAE	RRSE	RMSE
LR	0.3896	0.32	1.1245	1.1447	8.1507
RF	0.3721	0.277	1.0045	0.9818	6.9913
SGB	0.3728	0.2915	1.0495	0.97	6.9069
XGboost	0.3703	0.2816	1.0316	1.0679	7.6038
Elastic net	0.3579	0.2721	0.9805	0.9736	6.9325

LR: linear regression, RF: random forest, SGB: stochastic gradient boosting, NB: naïve Bayes, XGBoost: extreme gradient boosting.

**Table 5:** The importance, mean, and rank of the risk factors derived from linear regression and machine learning methods.

Variable	Linear	RF	SGB	XGBoost	Elastic net	Mean	MROI
Age	77.7	100.0	100.0	51.2	4.6	63.9	1.0
Plasma insulin level	58.1	95.1	47.2	100.0	4.3	61.6	2.0
Thyroid-stimulating hormone	58.1	80.7	49.9	73.0	15.8	54.8	3.0
Spouse status	100.0	33.1	27.8	35.5	100.0	49.1	4.0
Lactic dehydrogenase	79.8	91.7	51.8	49.3	1.6	48.6	5.0
Alkaline phosphatase	41.0	89.4	25.7	75.2	0.0	47.6	6.0
LDL-cholesterol	0.9	91.0	30.9	66.1	0.0	47.0	7.0
High-sensitivity CRP	34.2	81.0	66.6	39.3	0.0	46.7	8.0
HDL-cholesterol	90.5	84.9	67.0	30.0	2.7	46.2	9.0
Diastolic blood pressure	27.4	78.4	36.2	52.5	0.7	42.0	10.0
Alfa-fetoprotein	61.0	84.1	34.4	33.0	10.9	40.6	11.0
Glycated hemoglobin	52.7	71.0	0.0	24.4	61.2	39.1	12.0
Estimated glomerular filtration rate	3.7	92.5	21.1	38.0	0.0	37.9	13.0
FEV1	25.7	73.4	9.5	52.1	0.0	33.7	14.0
Uric acid	68.8	67.5	34.0	12.4	20.3	33.5	15.0
White blood cell count	21.5	73.0	15.5	43.1	0.0	32.9	16.0
Platelet cell count	25.9	73.2	35.8	21.4	0.0	32.6	17.0
Glutamic oxaloacetic transaminase	10.1	67.5	34.0	24.9	0.0	31.6	18.0
Plasma ferritin level	59.3	80.2	18.3	22.0	0.2	30.2	19.0
Body fat percentage	29.4	70.7	0.0	45.7	0.0	29.1	20.0
Carcinoembryonic antigen	22.0	68.1	0.0	44.5	0.0	28.1	21.0
Hemoglobin level	79.2	66.0	0.0	22.3	24.2	28.1	22.0
Free-testosterone level	49.7	66.9	9.4	28.9	2.1	26.8	23.0
Triglyceride	58.1	65.4	19.6	20.3	0.1	26.3	24.0
Systolic blood pressure	10.2	75.5	0.0	17.0	0.1	23.2	25.0
Sport hours/day	11.1	41.1	9.4	38.9	0.0	22.3	26.0
Total bilirubin	79.3	65.1	0.0	17.3	0.0	20.6	27.0
Plasma phosphate level	6.1	57.9	0.0	23.1	0.0	20.2	28.0
γ-Glutamyltransferase	30.2	61.9	0.0	17.8	0.0	19.9	29.0
Glutamic pyruvic transaminase	40.8	66.9	0.0	9.5	0.0	19.1	30.0
Family income/year	59.2	33.6	11.0	4.8	9.0	14.6	31.0
Plasma calcium level	11.9	44.2	0.0	8.8	0.0	13.2	32.0
Sleep hours/day	0.0	43.6	0.0	1.3	0.0	11.2	33.0
Education level	21.5	26.5	0.0	0.0	6.7	8.3	34.0
Betel nut	0.0	0.0	0.0	0.0	0.0	0.0	35.0

LR: linear regression, RF: random forest, SGB: stochastic gradient boosting, NB: naïve Bayes, XGBoost: extreme gradient boosting, eGFR: estimated glomerular filtration rate, MROI: mean rank of importance. FEV1: forced expiratory volume in one second.

## Data Availability

Written informed consent has been obtained from the represented patient(s) to publish this paper.
